# Estimating biomass and soil carbon change at the level of forest stands using repeated forest surveys assisted by airborne laser scanner data

**DOI:** 10.1186/s13021-023-00222-4

**Published:** 2023-05-20

**Authors:** Victor F. Strîmbu, Erik Næsset, Hans Ole Ørka, Jari Liski, Hans Petersson, Terje Gobakken

**Affiliations:** 1 Faculty of Environmental Sciences and Natural Resource Management, Norwegian University of Life Sciences, 1432 Ås, Norway; 2 Climate System Research, Finnish Meteorological Institute, 00101 Helsinki, Finland; 3 Department of Forest Resource Management, Swedish University of Agricultural Sciences, 901 83 Umeå, Sweden

**Keywords:** Forest carbon pools, Carbon change estimation, Forest inventory, Forest management, Model-based estimation, Soil carbon, Forest biomass, Yasso15

## Abstract

**Background:**

Under the growing pressure to implement mitigation actions, the focus of forest management is shifting from a traditional resource centric view to incorporate more forest ecosystem services objectives such as carbon sequestration. Estimating the above-ground biomass in forests using airborne laser scanning (ALS) is now an operational practice in Northern Europe and is being adopted in many parts of the world. In the boreal forests, however, most of the carbon (85%) is stored in the soil organic (SO) matter. While this very important carbon pool is “invisible” to ALS, it is closely connected and feeds from the growing forest stocks. We propose an integrated methodology to estimate the changes in forest carbon pools at the level of forest stands by combining field measurements and ALS data.

**Results:**

ALS-based models of dominant height, mean diameter, and biomass were fitted using the field observations and were used to predict mean tree biophysical properties across the entire study area (50 km^2^) which was in turn used to estimate the biomass carbon stocks and the litter production that feeds into the soil. For the soil carbon pool estimation, we used the Yasso15 model. The methodology was based on (1) approximating the initial soil carbon stocks using simulations; (2) predicting the annual litter input based on the predicted growing stocks in each cell; (3) predicting the soil carbon dynamics of the annual litter using the Yasso15 soil carbon model. The estimated total carbon change (standard errors in parenthesis) for the entire area was 0.741 (0.14) Mg ha^−1^ yr^−1^. The biomass carbon change was 0.405 (0.13) Mg ha^−1^ yr^−1^, the litter carbon change (e.g., deadwood and leaves) was 0.346 (0.027) Mg ha^−1^ yr^−1^, and the change in SO carbon was − 0.01 (0.003) Mg ha^−1^ yr^−1^.

**Conclusions:**

Our results show that ALS data can be used indirectly through a chain of models to estimate soil carbon changes in addition to changes in biomass at the primary level of forest management, namely the forest stands. Having control of the errors contributed by each model, the stand-level uncertainty can be estimated under a model-based inferential approach.

## Background

Forest management planning is traditionally focused on sustainable utilization of wood resources whereas other market-based or non-market-based services have received less attention. However, over the last 20–30 years, many efforts have been invested in accommodating other services in quantitative and consistent long-term strategic forest management analysis. Several recent studies have explored the trade-offs between resource extraction, ecosystem services and biodiversity in production forests, where timber harvesting and other forestry-related activities will tend to affect ecosystem structures and functions [1–10].

Among the United Nations’ Sustainable Development Goals, climate change mitigation received much attention, and in particular due to the momentum created by the Paris Agreement [11]. To stabilize the global temperature possibly below 1.5 °C above pre-industrial levels, large contributions across all economic sectors including agriculture and forestry is required [12–14]. This has direct implications for the required land-based mitigation efforts. Measures to avoid loss and to increase uptake in all carbon pools are viewed as essential [15]. Furthermore, under the current legislation of the European Union (EU) adopted in May 2018, EU Member States must ensure that accounted greenhouse gas emissions from land use, land use change or forestry sector are balanced by at least an equivalent accounted removal of CO_2_ from the atmosphere in the period 2021 to 2030 [16]. In October 2020, the EU Commission amended the existing Land Use, Land-Use Change and Forestry (LULUCF) legislation with a delegated act [17] setting forest reference levels that each country must apply between 2021 and 2025.

The United Nations Framework Convention on Climate Change (UNFCCC) has included five carbon pools for estimating the impacts of land-use change and forestry activities: above-ground biomass, below-ground biomass, dead wood, litter, and soil organic matter [18], which is reflected in IPCC [19] technical guidance for greenhouse gas inventories. Soil is the largest terrestrial carbon reservoir [20] and a major source of uncertainty in ecosystem carbon predictions [21]. Boreal forest ecosystems account for approximately 50%, or more, of the global forest carbon stocks [22]. Furthermore, boreal forest soils hold more carbon compared to the overstory [23–26]. Indeed, soil carbon in boreal ecosystems has been reported to account for about five times the total carbon in the standing biomass or approximately 85% of the total biome carbon [22]. Yet, soil carbon pools are rarely considered in those decisions that affect the climate impact of the forests the most, namely the daily management of forests across the entire boreal zone. Quantification of soil carbon pools and their changes at a local level where practical management decisions are implemented in the form of harvesting, thinning, tending and other actions, is also difficult and costly, whereas data and methodologies for quantification of other pools such as living aboveground biomass, are extensively investigated and described [27], but still not commonly taken into account in the actual management of forests.

Reliable estimation of changes in different forest carbon pools has for several reasons become a prominent issue in forest inventory at a broad range of geographical scales. At local levels, forest management inventories conducted for individual forest estates or for groups of estates within an administrative area, are in many cases the most reliable source of information on forest resources and carbon stocks. Such inventories are often designed to provide cost-effective estimates of current timber resources and are less optimized for future monitoring of changes. However, with the methodology already established in such local or district-wise inventories it may provide an advantageous option for measurement and verification of carbon offset activities or local monitoring of carbon stocks. The individual forest stands are usually considered the basic treatment units under management regimes currently adopted across the boreal forests (cf. [28]). This geographical unit is therefore fundamental when addressing carbon pools and how they are affected under practical management.

Various remote sensing technologies have been used extensively to estimate forest resources. Airborne laser scanning (ALS) data has high spatial resolution and is rich in information on vertical structure of above-ground vegetation, which is why it has emerged as one of the best suited and cost-effective remote sensing technologies for estimating above-ground tree biomass and carbon stocks. Studies of biomass change estimation with ALS started to emerge with the opportunities created by repeated acquisitions at either local [29–33], regional [34, 35] or cross-regional scales [36]. In some countries, local forest management inventories assisted by ALS have over the past two decades become the main methodology for stand-wise estimation of forest attributes needed for forest management planning [37]. Use of bi-temporal data from ALS has recently been adopted for some of the time-dependent attributes needed in the planning process, such as site productivity reflecting growth potential over time [38]. Næsset et al. [32] demonstrated how areal changes for different categories of management activities and associated changes in above ground biomass can be estimated by repeated measurements of a sample of field plots supported by coincident and repeated measurements with ALS. However, there is little evidence in existing literature on how soil carbon can be estimated at the stand level for management purposes based on sparse and non-destructive sampling on the ground, possibly combined with commonly adopted remotely sensed data, such as those acquired by ALS. Soil accumulation is highly dependent on the local topography [39, 40], and Kristensen et al. [41] showed that while ALS derived topographic indexes are good predictors for soil carbon stocks, above-ground ALS metrics and even forest characteristics measured in the field did not relate well with the soil pool. Local topography corroborated with rainwater [42, 43] move the soil carbon away from the production site which makes it difficult to isolate the carbon balance of an individual forest stand using only carbon stocks measured or predicted spatially. Hopkinson et al. [44] proposed to estimate changes in soil carbon by subtracting ALS based biomass changes from the total flux measured atmospherically. This approach while promising on larger scales, would be difficult to operationalize at the stand level due to high costs and interference of atmospheric flux from neighboring stands.

To be able to set targets on e.g. the magnitude of carbon stored in different pools at a stand level and to estimate expected changes in different pools over time as a consequence of different active treatments, there is a need for (1) inventory methods and estimation techniques to quantify the initial magnitude of the carbon pools and (2) for methods to estimate changes over time in the past and future as a result of prescribed treatment actions. Akujärvi et al. [45] proposed a framework to map the future development of carbon stocks in biomass and soil by simulating the effect of future silvicultural treatments. In the present study, based on similar principles that link the living biomass to the soil carbon accumulation, our primary objective was to develop an integrated stand-level methodology to estimate the carbon change in five forest pools using field plots with coincident and repeated ALS measurements. To reveal the benefit of the magnitude of carbon stored in different pools at a stand level and to estimate expected changes in different pools over time as a consequence of different active treatments [46] we demonstrate the methodology in a case study of a Norwegian forest.

## Materials and methods

### Materials

#### Study area

**Fig. 1:**
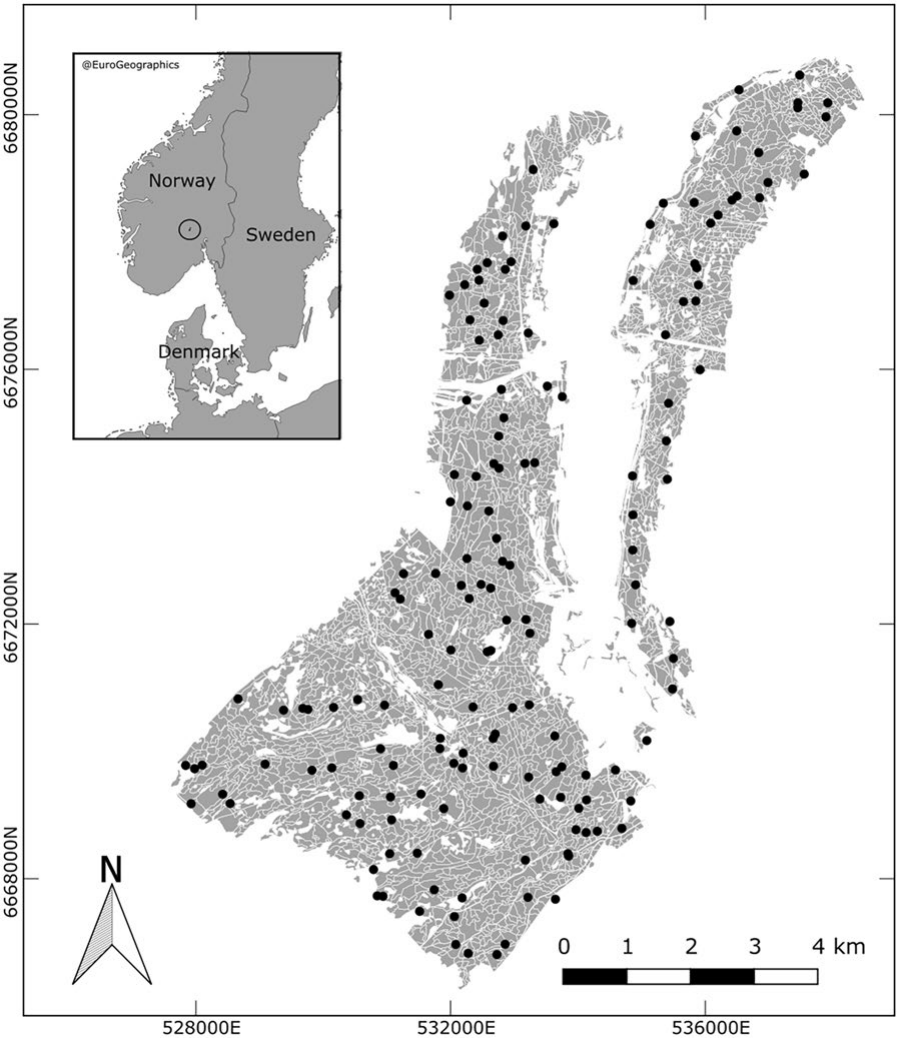
Study area. The field plot locations are marked with black dots

The study area (Fig. 1) lies in Krødsherad municipality, southeastern Norway. It is a typical boreal forest dominated by Norway spruce [*Picea abies* (L.) Karst.], Scots pine (*Pinus sylvestris* L.), and, to a less extent, birch species (*Betula pendula* Roth and *Betula pubescens* Ehrh). The forested area consists of 3324 managed forest stands spanning approximately 50 km^2^.

#### Field data

A total of 116 circular area plots (232.9 m^2^) were distributed systematically within three strata: young forest (39 plots), mature forest with poor site quality (38 plots, site index ≤ 11), and mature forest with good site quality (39 plots, site index > 11). The plots were measured in two campaigns, approximately 15 years apart. The first campaign was conducted in 2001 followed by a second when all plots were revisited in 2016 and 2017. For the second campaign that spanned two years, 2016 was set as reference year. The diameters of all trees above 10 cm (4 cm in the young forest stratum) were measured with a caliper, and the heights of a relascope-based subsample of around 10 trees per plot were measured with a hypsometer. The following variables were calculated: mean diameter ($D$), mean height ($H$), dominant height (${H}_{dom}$), and number of trees per hectare ($N$). Using allometric models [47], total living biomass per hectare ($BMS$) was estimated. All sample plot variables were assumed to be without error.

#### Airborne laser scanner data

The ALS acquisition temporally overlapped the field data collection, the entire study area having been scanned under leaf-on conditions in 2001 and 2016. The field surveys and ALS acquisitions are described in greater detail in [48] and [49].

The ALS point clouds were normalized and tessellated with a grid of 15.26 m $\times$ 15.26 m cells having the same area as the field plots. For each cell, metrics describing the vertical distribution of the laser returns were computed:

- Height percentiles ${H}_{ALS\_10}$, ${H}_{ALS\_20}$, …, ${H}_{ALS\_90}$ corresponding to the 10th, 20th, …, 90th percentiles
- Mean height ($${H}_{ALS\_mean}$$)
- Cumulated densities ${D}_{ALS\_1}$, ${D}_{ALS\_2}$, …, ${D}_{ALS\_10}$ as proportions of points above ten equally spaced height levels from 1.3 m to the 95th height percentile.

The set of ALS metrics were calculated for the 116 georeferenced sample plots as well.

#### Forest stand map

**Fig. 2:**
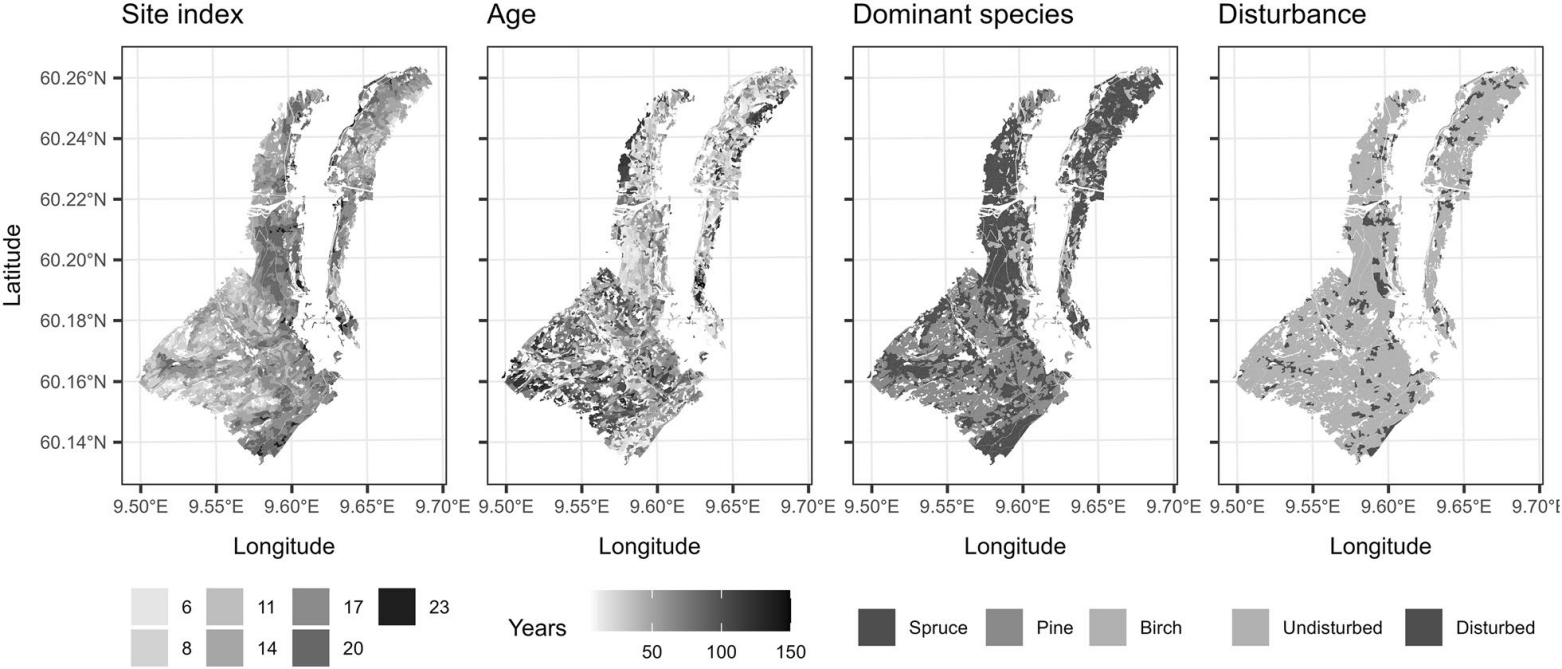
Forest stand attributes

A forest stand map was obtained from the forest management inventory carried out in the area in 2018. Each stand had the following attributes: site index ($SI$), stand age ($AGE$), and species proportions by volume recorded, which determined the dominant species ($SP$). The stand attributes (Fig. 2) were obtained using a combination of projections of the old stand map and updates using photointerpretation of aerial imagery.

#### Satellite imagery

Landsat satellite imagery were used to detect large disturbances, which were assumed to be the result of harvest or thinning. We used the temporal segmentation algorithm LandTrendr [50], implemented with Google Earth Engine [51]. LandTrendr has been widely used and shows good performance in similar environments [52]. A disturbance map was created based on the ALS tessellation and recording the year of disturbance.

#### Tree allometry dataset

Marklund’s [47] allometric models have been extensively employed in Norway and Sweden for many decades. They are used to predict biomass for individual tree components. The original publication reported the estimated model parameters but did not include any materials on the errors associated with the parameters. Using the original set of observations, we refitted the models and estimated the covariance matrices for the parameters, which are needed for the statistical inference.

The dataset consisted of 1281 individual trees (546 spruce, 494 pine, and 241 birch) with measured heights and diameters. The response variables were biomass of stem wood ($SW$), branches ($BR$), dead branches ($DB$), bark ($SB$), stump ($SU$), foliage ($FL$), fine roots ($RF$), and coarse roots ($RC$). The number of observations for each biomass component varies as shown in Table 1.

**Table 1:** Number of observations for each biomass component by species. (Data from Marklund’s 1988 allometric models [47])

	$SW$	$BR$	$DB$	$SB$	$SU$	$FL$	$RF$	$RC$
Spruce	521	540	525	521	323	540	324	333
Pine	471	486	472	471	305	485	305	314
Birch	218	235	221	218	0	0	0	0

#### Climate data

The climate data for this study have been acquired using the Frost API of the Norwegian Meteorological Institute [53]. The data are licensed under Norwegian license for public data (NLOD) and Creative Commons 4.0 BY. Historical weather data were retrieved by identifying the nearest weather station and retrieving timeseries of daily precipitation and temperature data. For each year from 2001 to 2016 the following climate variables were calculated: annual precipitations (mm), mean annual temperature (°C) and the mean difference between maximum and minimum monthly temperatures (°C). Another set of climate variables was calculated with data starting from 1957, the earliest recorded year to 2000. For these older observations, the three variables were averaged across the 43 years.

### Methods

#### Overview

We start with a brief overview of the proposed methodology. First, we introduce the five variables of interest to be estimated at the stand level: ${\Delta C}_{AGB}$, ${\Delta C}_{BGB}$, ${\Delta C}_{litter}$, ${\Delta C}_{deadwood}$, and ${\Delta C}_{SOC}$. They represent the change in carbon mass expressed in Mg ha^−1^ yr^−1^ within the following pools: above-ground biomass (AGB), below-ground biomass (BGB), litter, deadwood, and soil organic (SO) carbon.

We use an indirect method to estimate change [31], by taking the difference between estimates of C stocks at the start and end of the time period (i.e., ${\Delta C}_{AGB}=\frac{{C}_{AGB|2016}-{C}_{AGB|2001}}{2016-2001}$). Moreover, in lack of ground observations we rely on a model-based approach where the inference is based on the properties of the models involved in the estimation [54, 55].

The carbon stocks in the living biomass pools (${C}_{AGB}$ and ${C}_{BGB}$) were estimated for both points in time using area-based ALS models. The carbon stocks in the dead biomass pools (${C}_{deadwood}$ and ${C}_{litter}$) and ${C}_{SO}$ pools, however, are accumulations sourced from the biomass pools that go through a decomposition process. To estimate their levels, we need to: (1) approximate the litter and deadwood production, and (2) simulate the decomposition process. For the first part, we calculated the litter production based on the living biomass stocks and active silvicultural treatments. We considered three mechanisms that generate litter: annual litter turnover, annual mortality, and excess litter resulting from harvest. The decomposition process was simulated using the Yasso15 soil model [56].

The estimation processes involve several models (see “Methods/Models” section) that are linked together in a chain of predictions (see *Methods/Estimation process* section), the outcome being carbon stocks in the five pools at cell level. For the soil-related pools (${C}_{deadwood}$, ${C}_{litter}$, and ${C}_{SO}$) the carbon stocks are calculated year by year starting from 2001, through 2016, each time carrying through the accumulated carbon from the previous year and integrating new yearly litter.

The stand level estimates of carbon change in each pool are obtained by averaging predictions over the cells within each stand. To estimate the uncertainty, we used a Monte Carlo approach, by sampling repeatedly from the models’ parameters distributions using their estimated means and covariance matrices and assuming joint normal distributions (see “Methods/Estimation process/Model based estimation and uncertainty” section).

### Models

#### Yasso15 model

**Fig. 3:**
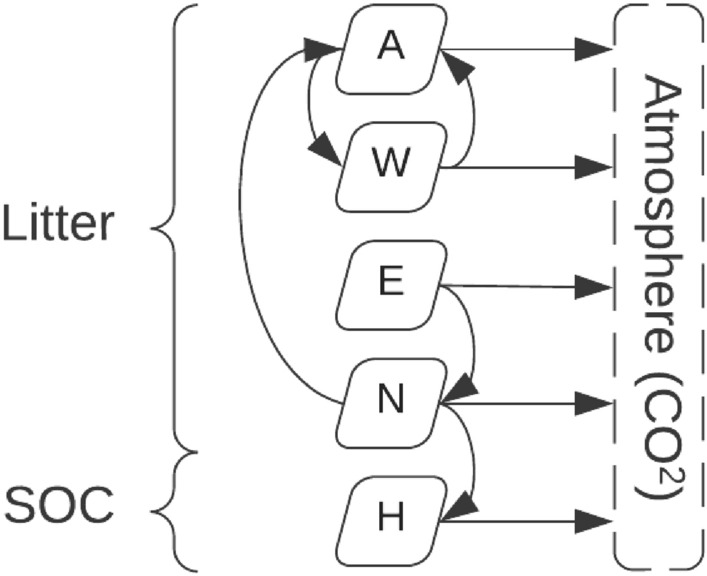
Carbon flux diagram of the Yasso15 soil model. The abbreviations are: soil organic carbon ($SOC$), celluloses ($A$), sugars ($W$), wax-like compounds ($E$), lignin-like compounds ($N$), and humus ($H$)

The Yasso15 model [56] partitions soil in five chemical compartments. Four of these compartments belong to the decomposing litter: celluloses ($A$), sugars ($W$), wax-like compounds ($E$), and lignin-like compounds ($N$). The fifth compartment is humus ($H$) as the end of the decomposition process. The carbon accumulated in humus makes up the soil organic (SO) carbon pool. The Yasso15 model is formulated in terms of decomposition rates (Fig. 3). Each litter compartment decomposes to either: another litter compartment, humus, or is emitted as CO_2_. The carbon in the humus compartment is only emitted as CO_2_. The decomposition rates depend on three climate variables: annual precipitations (mm), mean annual temperature (°C) and the mean difference between maximum and minimum monthly temperatures (°C).

Since the flow rates of the Yasso15 model depend only on the climate variables, it allows to separate carbon from different sources and trace their decomposition independently. We used this property to separate the decomposition of the initial carbon stocks (e.g., stocks at the beginning of the timeframe) from the new carbon being stored from the litter produced during the timeframe of interest. Also, we treated separately carbon that originates from normal turnover and harvest (${C}_{litter}$) from carbon sourced in mortality (${C}_{deadwood}$).

#### Allometric models

The allometric models for tree component biomass are based on Marklund [47]. The models were refitted using the original set of field observations and the parameter covariance matrix was estimated together with the mean values. The models are of three forms:

1 $$\mathrm{ln}\left(comp\right)={\beta }_{0}+{\beta }_{1}\frac{D}{D+k}$$

2 $$\mathrm{ln}\left(comp\right)={\beta }_{0}+{\beta }_{1}\frac{D}{D+k}+{\beta }_{2}\mathrm{ln}(H)$$

3 $$\mathrm{ln}\left(comp\right)={\beta }_{0}+{\beta }_{1}\frac{D}{D+k}+{\beta }_{2}H+{\beta }_{3}\mathrm{ln}\left(H\right)$$

where $comp$ is one of: $SW$, $BR$, $DB$, $SB$, $SU$, $FL$, $RF$, $RC$.

Table 2 shows which model form was used for each component and species.

**Table 2:** Summary of the allometric models for biomass components

	$SW$	$BR(+FL)$	$DB$	$SB$	$SU$	$FL$	$RF$	$RC$
Spruce	Equation 3, k = 14	Equation 3, k = 13	Equation 3, k = 18	Equation 3, k = 15	Equation 1, k = 17	Equation 2, k = 12	Equation 1, k = 12	Equation 1, k = 8
Pine	Equation 3, k = 14	Equation 2, k = 10	Equation 3, k = 10	Equation 2, k = 16	Equation 1, k = 15	Equation 3, k = 7	Equation 1, k = 10	Equation 1, k = 9
Birch	Equation 2, k = 11	Equation 1, k = 10	Equation 3, k = 30	Equation 2, k = 14	Use pine	$\frac{0.011}{0.52}\times SW$	$\frac{0.042}{0.52}\times SW$	$\frac{0.042}{0.52}\times SW$

The k values are used in Eq. 1, 2, 3

#### Area based models

The area-based models were fitted using the 116 field plots for which both field-calculated biophysical variables and ALS metrics were available. All area-based models were selected using the Bayesian information criterion, restricting the maximum number of parameters to five and the maximum variance inflation factor to five. The models were time-invariant [30], fitted on observations from both points in time, and using a dummy variable: $T=0$ for 2001, and $T=1$ for 2016. The models had the following form:

Mean diameter model: $\mathrm{ln}\left(D\right)={\beta }_{0}+{\beta }_{1}{D}_{ALS\_4}+{\beta }_{2}{H}_{ALS\_mean}+{\beta }_{3}T$

Dominant height model: ${H}_{dom}={\beta }_{0}+{\beta }_{1}{D}_{ALS\_2}+{\beta }_{2}{D}_{ALS\_8}+{\beta }_{3}{H}_{ALS\_80}+{\beta }_{4}T$

Biomass model: $$\mathrm{ln}(BMS)={\beta }_{0}+{\beta }_{1}{D}_{ALS\_2}+{\beta }_{2}{H}_{ALS\_80}+{\beta }_{3}T$$

Based on the same dataset a simple model was fitted to predict mean tree height ($H$) using ${H}_{dom}$:

Mean height model:$H={\beta }_{0}+{\beta }_{1}{H}_{dom}+{\beta }_{2}T$

We needed to predict both mean and dominant height since the growth models work with dominant heights and the biomass component models are for individual trees, thus using the mean height.

#### Models with unaccounted uncertainty

There are several external models for which the authors published only the mean parameter estimates, and thus we could not account for the uncertainty in the estimated parameters.

The diameter growth models published by Blingsmo [57] were fitted on plot data from the Norwegian national forest inventory (NFI). The growth period was on average five years, and the number of observed periods for each species was: 1385 for spruce, 1292 for pine, and 662 for birch. The diameter growth models were fitted separately for each species with the following dependent variables: diameter ($D$), site index ($SI$), number of trees per hectare ($N$), dominant height (${H}_{dom}$), and age ($AGE$).

The dominant height growth models were published by Sharma et al. [58] for spruce and pine and Eriksson et al. [59] for birch. The dependent variables were $SI$ and $AGE$.

The litter turnover rates and the AWEN partition of tree biomass components were used as fixed values, with unaccounted uncertainty. The carbon content of biomass was also a constant, i.e., 50%.

### Estimation process

**Fig. 4:**
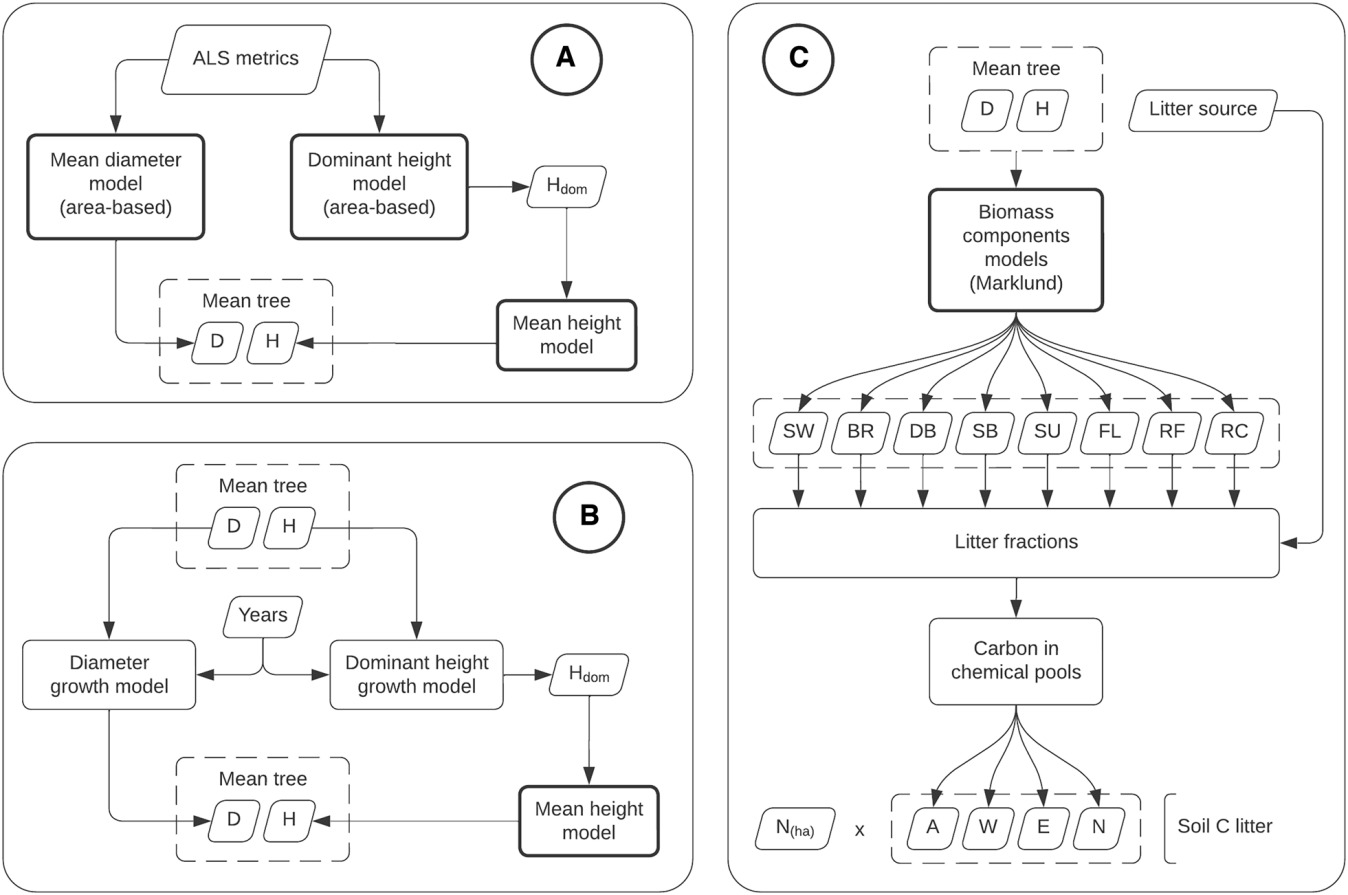
Models overview. **A** mean tree prediction, **B** growth models, **C** litter calculation. Bolded boxes are models with estimated parameter uncertainties. The symbols are: $D$ mean diameter, $H$ mean diameter, ${H}_{dom}$ dominant height, ${N}_{ha}$ number of trees per hectare, $SW$, $BR$, …, $RC$ are biomass of individual tree components (see “Methods/Models/Allometric models” section); $A$, $W$, $E$ and $N$ are carbon in chemical compartments (see “Methods/Models/Yasso15 model” section)

**Fig. 5:**
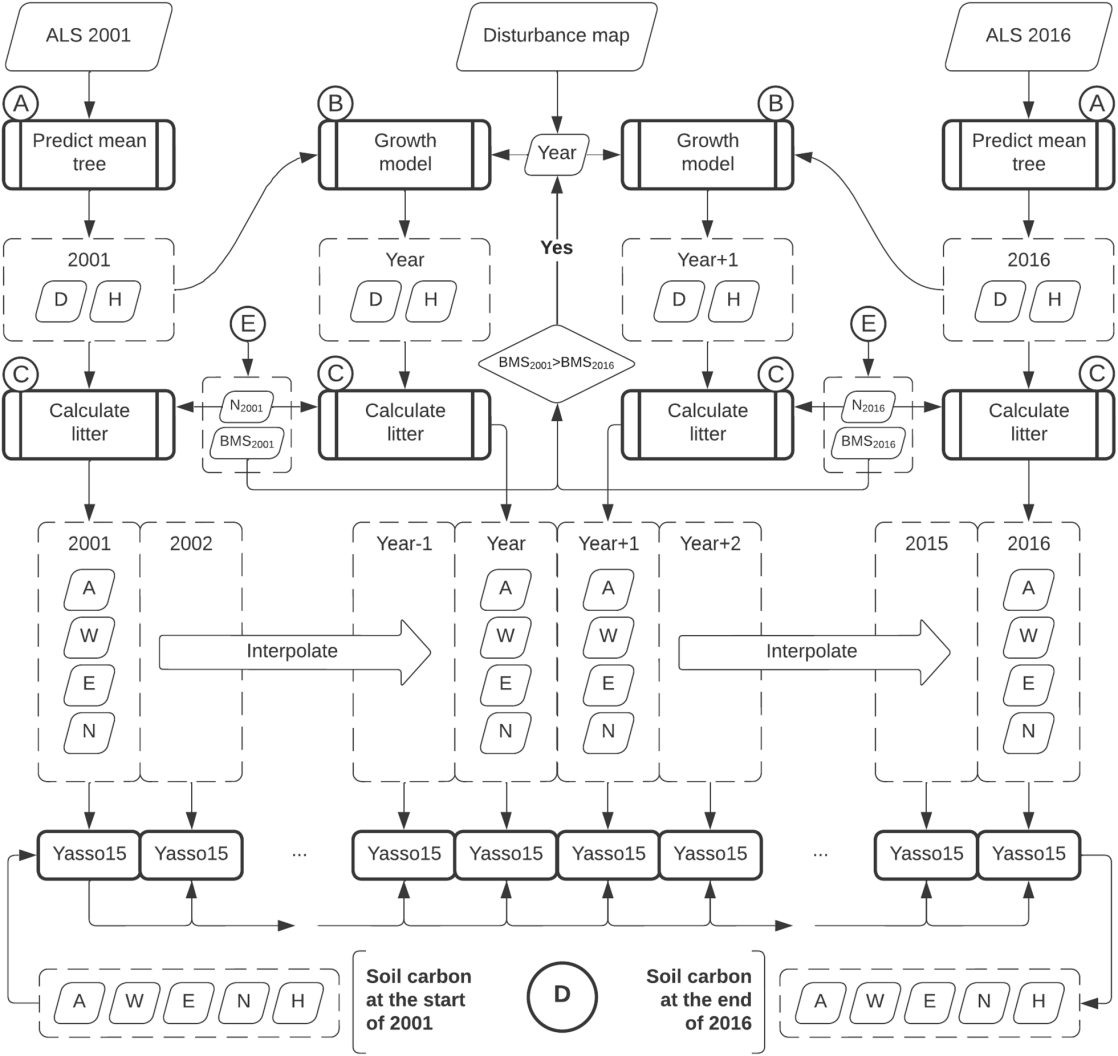
Models overview. Soil Carbon change computation within the 15-year timeframe. Bolded boxes are models with estimated parameter uncertainties. **E** Links to the process in Fig. 6

**Fig. 6:**
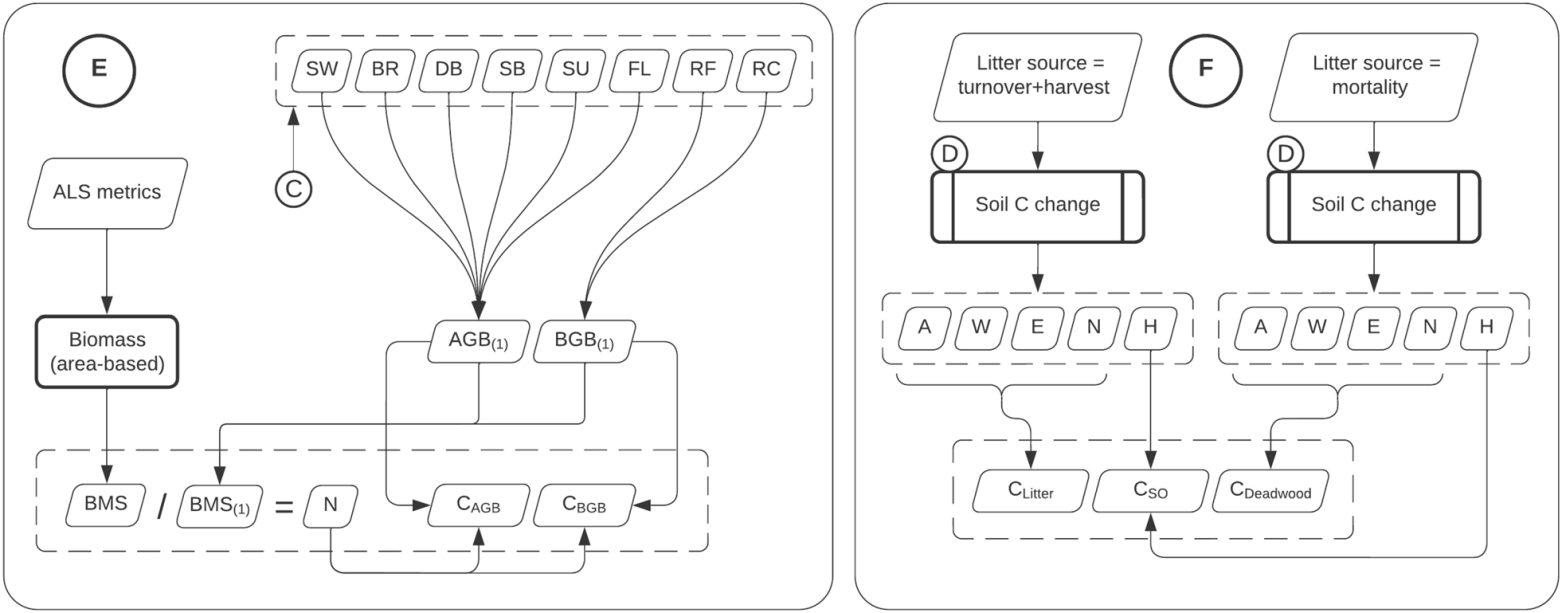
Models overview. **E** Predicting stocks of above-ground biomass carbon (${C}_{AGB}$), below-ground biomass carbon (${C}_{BGB}$) and number of trees per hectare ($N$). **F** Predicting litter Carbon (${C}_{litter}$), deadwood Carbon (${C}_{deadwood}$) and soil organic carbon (${C}_{SO}$). Bolded boxes are models with estimated parameter uncertainties

Computing the estimated change in soil and biomass carbon involved combining several models including ALS area-based models, allometric models, growth models and the Yasso15 soil model which were described in the previous section. For a better overview, we grouped the models in several functional blocks (Fig. 4). The core process is illustrated in Figs. 5 and 6 shows how the final predictions for each of the five pools are calculated.

The mean tree in each cell is characterized by its diameter ($D$) and height ($H$), and it was predicted using ALS area-based models (Fig. 4A). $H$ was predicted in two steps, with the dominant height model as intermediary. The growth models are illustrated in Fig. 4B. Again, $H$ is obtained via ${H}_{dom}$.

Finally, Fig. 4C illustrates the litter calculation. Starting with the mean tree, the biomass components are predicted using the Marklund models. Next, depending on the litter source and tree species, a certain fraction of each component is retained as litter. We consider three litter sources: normal turnover, mortality, and harvest. The biomass fractions for the normal turnover are shown in Appendix A. For mortality, the fractions are 100% of all components, and in case of harvest, 95% of the stem wood is extracted (i.e., 5% left as litter), while the rest of biomass components are left in the forest and turn to litter entirely (100%). The yearly mortality rate was determined by the difference in the estimated number of stems (see Fig. 6E) at the two points in time, and in the case of non-decreasing stem number a constant rate of 0.4% was assumed yearly [60].

Finally, the litter originated from each biomass component was partitioned into four compartments according to the chemical composition: $A$, $W$, $E$, $N$ (see Appendix B).

The core prediction process is illustrated in Fig. 5. It consists in estimating the mean tree at key points in time, calculating the litter associated with that, obtaining the yearly litter production by interpolating the litter quantities for the years in between, and finally use the Yasso15 model in a chain of predictions.

Since evolution of the growing stocks depends on whether the forest grew undisturbed or there was a disturbance event, we considered two scenarios: undisturbed forest growth, and disturbance detected. The forest was assumed to be disturbed if the estimated biomass from the ALS survey ($BMS$—area based) decreased between 2001 and 2016. In this case, the disturbance map would provide the approximate year of the disturbance event. If no year was recorded, then the event was assumed to have happened in the middle of the time interval. In the simpler case of undisturbed growth, the yearly litter was calculated by interpolating linearly between the litter quantities associated with the mean tree at the start and the end of the time interval. If disturbance was detected, two additional mean trees were predicted: the mean tree right before the event, and the mean tree right after the event. The “before” tree was predicted using the growth models with the tree in 2001, and the “after” tree was predicted using inverse growth models with the tree in 2016. As the growth models are difficult to invert analytically, an approximation search algorithm was used, where the search interval was recursively halved. The tolerance for both height and diameter predictions were set to be less than 10^–3^ m. In this scenario we have four points in time with determined growing stocks size, and corresponding litter production. We interpolate linearly for the years in between 2001 and the year of the event, and from the following year to the end in 2016. For the year of the disturbance event, we assume an active silvicultural treatment (thinning or harvest), and the normal litter production is supplemented by the vegetal residuals typically left on site (i.e., all but 95% of the stem wood).

The initial soil carbon quantities (at the start of the timeframe; 2001) denoted here as “old” soil carbon were approximated using simulations in two stages. First, the long-term soil carbon was calculated by running the model iteratively with a fixed litter input until the carbon quantities in the chemical compartments reach an equilibrium. The litter input was determined using average values of the growing stocks in 2001 for the entire study area separately for each tree species and site index (Table 3). In absence of empirical soil carbon observations or knowledge of the old history of the stand, this ensured that all stands with similar productivity have a common soil carbon baseline.

**Table 3:** Long-term soil carbon values (Mg ha^−1^) by species ($SP$) and site index ($SI$)

$SP$	$SI$	$A$	$W$	$E$	$N$	$H$
Spruce	6	6.97	0.73	0.68	17.74	35.09
	8	5.45	0.57	0.53	13.88	27.43
	11	7.10	0.75	0.69	18.07	35.77
	14	7.16	0.76	0.70	18.23	36.06
	17	7.94	0.84	0.78	20.21	39.99
	20	8.17	0.86	0.80	20.78	41.12
	23	8.36	0.88	0.82	21.29	42.11
Pine	6	4.56	0.48	0.51	11.33	23.02
	8	5.64	0.60	0.63	13.98	28.43
	11	7.35	0.78	0.81	18.21	37.04
	14	8.35	0.88	0.92	20.68	42.05
	17	9.98	1.05	1.09	24.73	50.28
	20	11.81	1.25	1.29	29.25	59.45
Birch	8	5.15	0.56	0.62	13.54	26.42
	11	7.34	0.79	0.90	19.33	37.66
	14	7.50	0.81	0.91	19.74	38.49
	17	8.67	0.94	1.06	22.84	44.49
	20	9.41	1.02	1.15	24.80	48.31
	23	14.06	1.52	1.74	37.16	72.27

In the second stage, the evolution of soil C was calculated for the current silvicultural cycle which was assumed to have started with a clear cut and the soil carbon values in Table 3. The stand $AGE$ in 2001 determined how many years have passed in the current silvicultural cycle. The soil carbon model was applied for each year in the current cycle until 2001, with the values for the yearly litter input being linearly interpolated between 0 and the ones calculated for the year 2001.

We expect the soil carbon initialization to be a coarse approximation of the soil carbons stocks in the year 2001, so we kept this value separated from the predicted accumulation during the timeframe that is based on several good quality data sources and models. This means that: (1) we traced the old soil carbon separately through the 15-year timeframe, with no yearly litter input, and (2) the soil carbon starts with 0 initial values for the within timeframe process (Fig. 5). Finally, the old carbon stocks in the year 2016 may be added to the new carbon accumulated in the timeframe, the result being identical to having the timeframe processing initialized with the old carbon in 2001.

The final steps to obtain carbon stocks in the five pools are shown in Fig. 6 (E—for AGB, and BGB; F—for litter, deadwood, and SO). The ${C}_{AGB}$ and ${C}_{BGB}$ stocks are calculated using the related mean tree biomass components (i.e., $RF$ and $RC$ belong to BGB and the rest to AGB), and then scaling up to per hectare values using the total $BMS$ predicted with the area-based model. The number of trees per hectare is calculated by dividing $BMS$ to the biomass of the mean tree. ${C}_{litter}$ and ${C}_{deadwood}$ are calculated separately using the process in Fig. 5. ${C}_{litter}$ accounts for the normal litter turnover plus the harvest residues, and ${C}_{deadwood}$ accumulates carbon sourced in mortality. ${C}_{litter}$ and ${C}_{deadwood}$ are calculated by summing up the carbon in the litter chemical compartments: $A+W+E+N$. Finally, ${C}_{SO}$ consists of the humus ($H$) compartments of both ${C}_{litter}$ and ${C}_{deadwood}$. Table 4 shows the connection between the Yasso15 chemical compartments, and the soil carbon pools.

**Table 4:** Summary of the Yasso15 chemical compartments, carbon input origin, and the soil carbon pools

	Timeframe		Historical	
	To. + Hv.	Mort.	To.	Mort.
A	${C}_{litter}$	${C}_{deadwood}$	${C}_{litter(old)}$	
W				
E				
N				
H	${C}_{SO}$		${C}_{SO(old)}$	

The abbreviations are turnover (To.), harvest (Hv.), and mortality (Mort.)

#### Model based estimation and uncertainty

As shown in the previous sections, predicting the soil carbon change in a cell involved the iterative use of the Yasso15 soil model and a series of interconnected models to calculate the yearly litter. The resulting soil carbon change predictions were then aggregated at the stand level. Since tracing the errors analytically would be extremely tedious, we used parametric bootstrapping [61]. This is a Monte Carlo-style method where the parameter values of each model were iteratively sampled from their estimated distributions, each time recalculating the whole chain of predictions to a new outcome. For the five models that we fitted ourselves (i.e., area-based biomass, dominant height, mean height, diameter, and biomass components—Marklund) we sampled from the joint parameter distributions defined by estimated means and variance–covariance matrices [62]. For Yasso15, the Finish Meteorological Institute provided us with a readily generated sample of 10,000 pairs of parameter values. For each parameter sample the entire sequence of predictions was recalculated to a different outcome (i.e., carbon change in the five pools). The errors were thus approximated by the sampling distribution of the change estimators.

The significance of the change estimates was assessed by calculating 95% confidence intervals based on the range of two standard errors. We report the number of stands for which the confidence interval did not include 0.

To assess the contribution of an individual model to the total error, we ran separate Monte Carlo simulations for each of the six models, where parametric bootstrapping was applied to the parameters of one model at a time, keeping the parameter of the rest of the models fixed at their estimated mean. This type of analysis does not account for the interaction between the models, the separate error components do not add up to the errors calculated for all models at once, so we report their relative size as percentage of the total error.

## Results and discussion

### Stand-level estimates

**Fig. 7:**
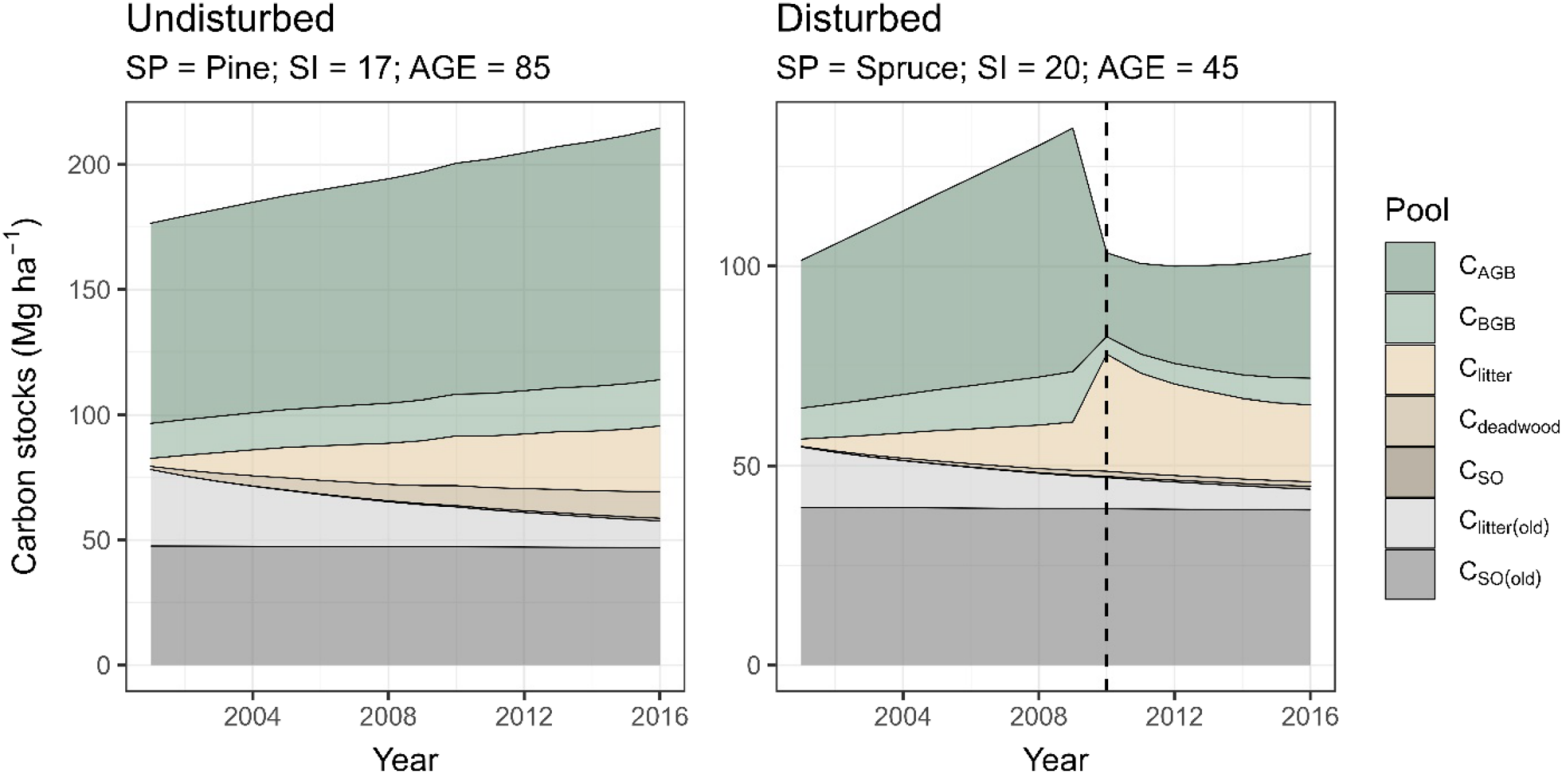
Yearly carbon stocks dynamics for two example forest stands: undisturbed (left), and disturbed (right). The year of disturbance (2010) is marked with a dashed line

To illustrate the carbon dynamics in a forest stand we plotted (Fig. 7) the estimated carbon stocks yearly between 2001 and 2016 for selected stands. The first stand (Fig. 7 left) was undisturbed while for the other (Fig. 7 right) a disturbance (likely a thinning) was detected in 2010. The old, accumulated litter carbon (${C}_{litter(old)}$) and SO carbon (${C}_{SO}$) are shown stacked in gray bands. Note how there is a sudden transfer from the biomass pools (${C}_{AGB}$ and ${C}_{BGB}$) to the litter pool in the disturbed stand. Then, in the years following the thinning, the biomass pools resume carbon accumulation while the litter pool ${C}_{litter}$ decrease as the new yearly production is at a lower level.

**Fig. 8:**
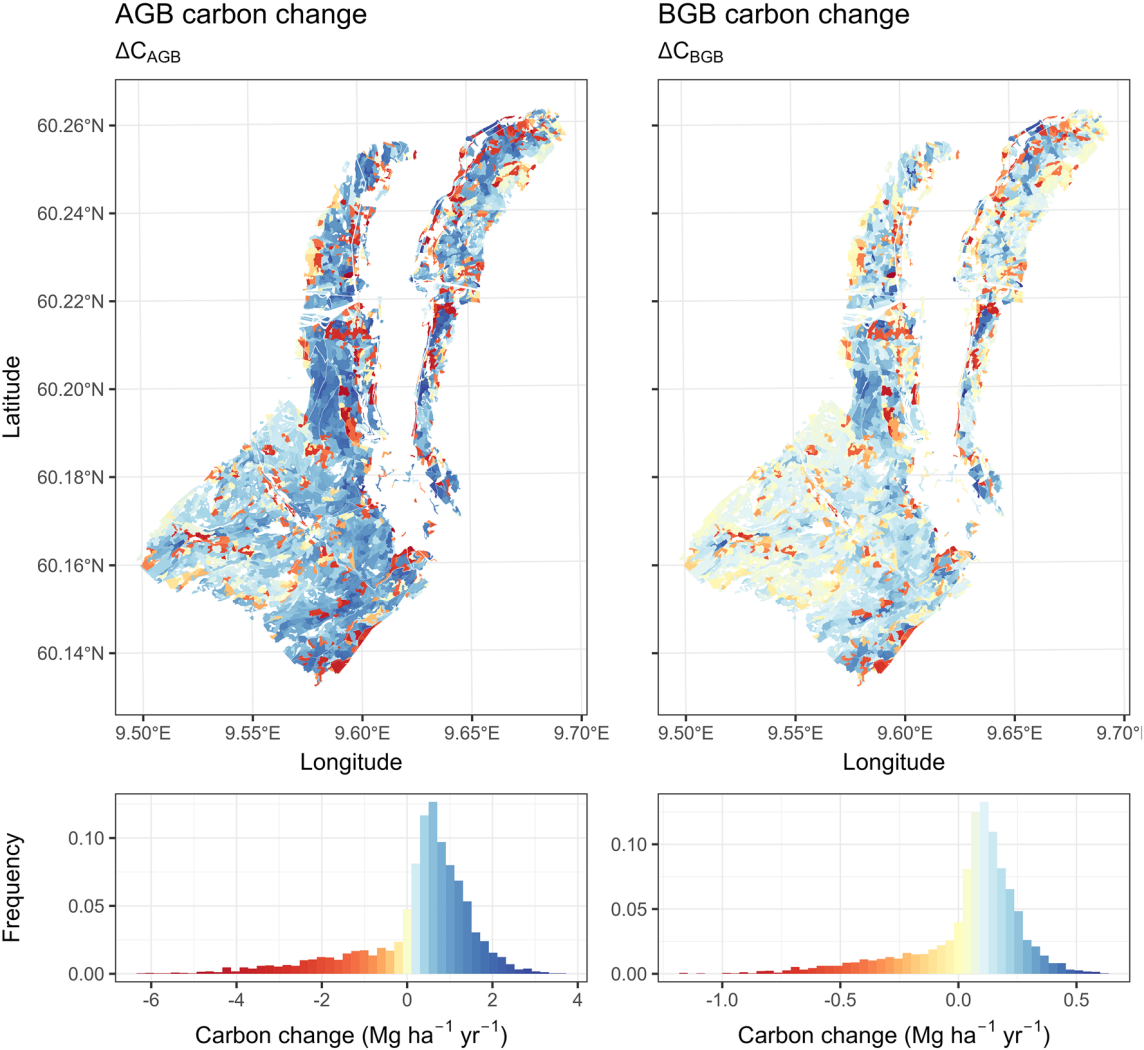
Estimated stand-level carbon change of the AGB and BGB pools

**Fig. 9:**
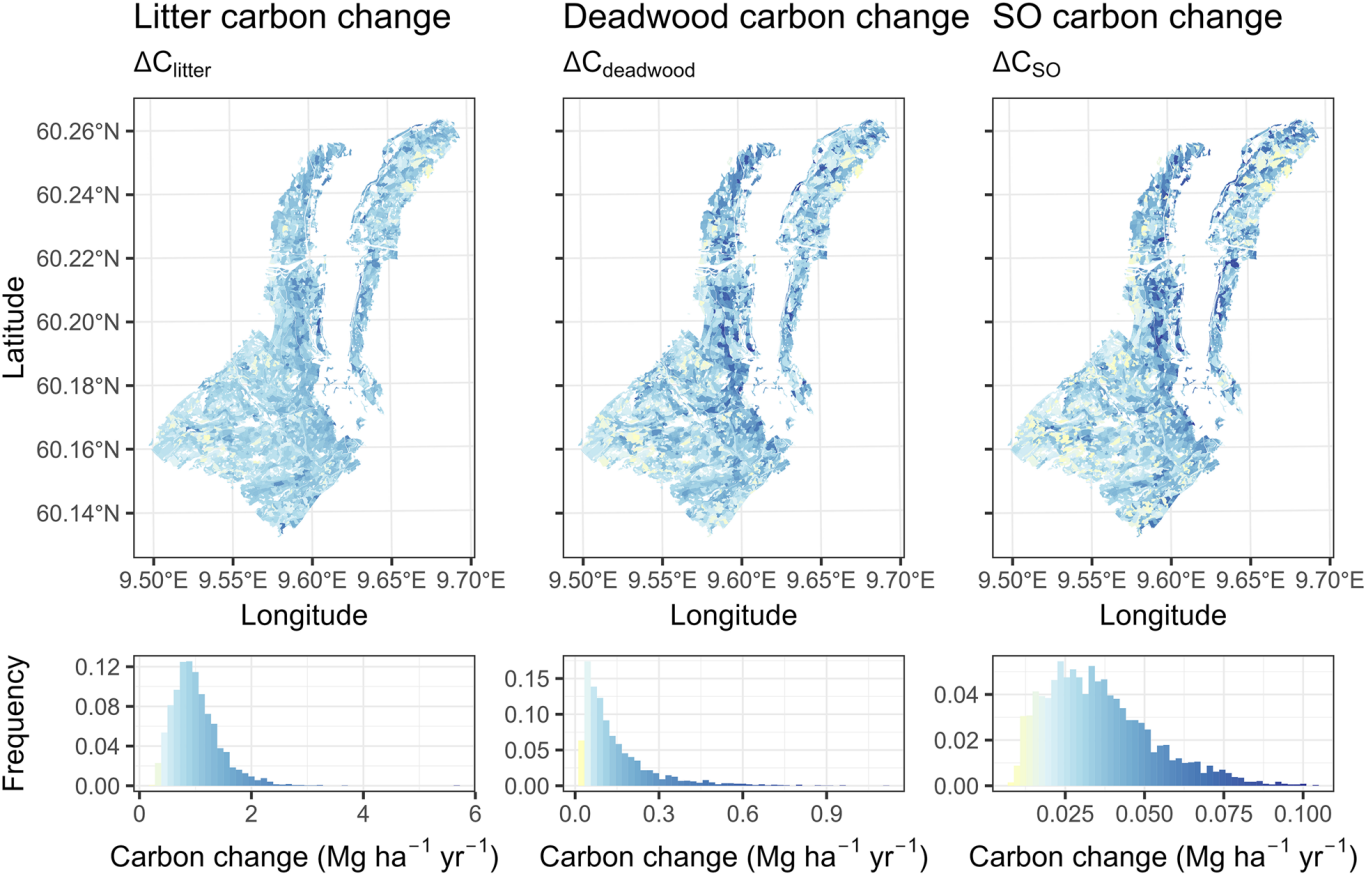
Estimated stand-level carbon change of the litter, deadwood, and SO pools

**Fig. 10:**
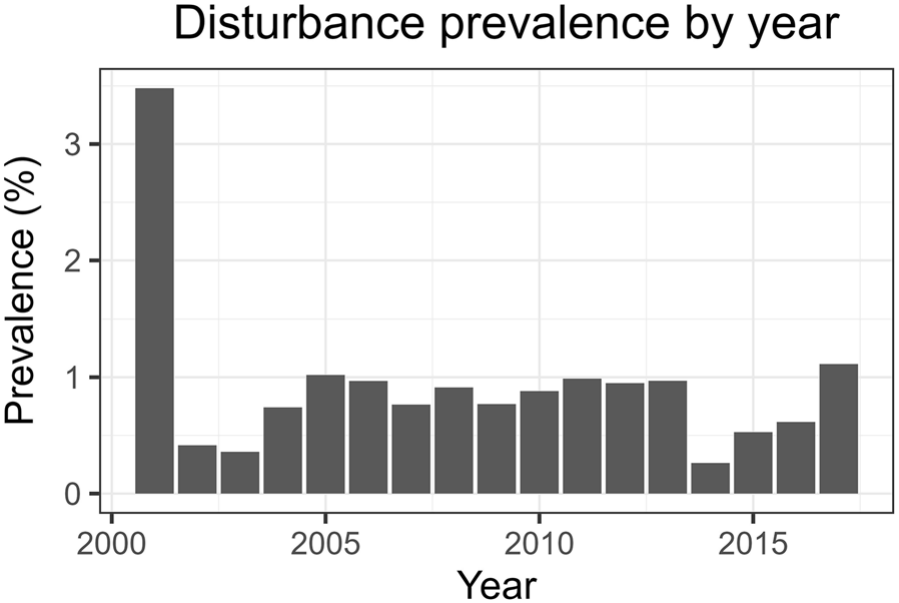
Disturbance year prevalence of the total study area. In total, 15.74% of the area was disturbed

The stand-level estimates of carbon change are shown on the map in Fig. 8 (${\Delta C}_{AGB}$ and $\Delta {C}_{BGB}$) and Fig. 9 (${\Delta C}_{litter}$, ${\Delta C}_{deadwood}$, and ${\Delta C}_{SO}$). ${\Delta C}_{AGB}$ ranged from − 6.196 Mg ha^−1^ yr^−1^ to 3.674 Mg ha^−1^ yr^−1^, with a mean change at 0.313 Mg ha^−1^ yr^−1^. ${\Delta C}_{BGB}$ followed a similar pattern, with stand-level estimates ranging from − 1.175 to 0.608 Mg ha^−1^ yr^−1^. On average ${\Delta C}_{BGB}$ was 0.056 Mg ha^−1^ yr^−1^. The observed skewed distribution of change is expected under typical forest management, where silvicultural treatments are continuously performed over the years. In this case study, within the 15-year timeframe, disturbance was detected on approximately 15.74% of the study area. Figure 10 shows the prevalence of disturbance by year.

The litter carbon accumulation (${C}_{litter}$) was estimated between 0.244 and 5.689 Mg ha^−1^ yr^−1^, with an average of 1.004 Mg ha^−1^ yr^−1^. The deadwood carbon accumulation (${C}_{deadwood}$) was between 0.014 and 1.105 Mg ha^−1^ yr^−1^, and 0.145 Mg ha^−1^ yr^−1^ on average. ${C}_{SO}$ accumulation ranged from 0.008 to 0.104 Mg ha^−1^ yr^−1^. On average ${C}_{SO}$ accumulated 0.036 Mg ha^−1^ yr^−1^ during the 15-year period.

**Fig. 11:**
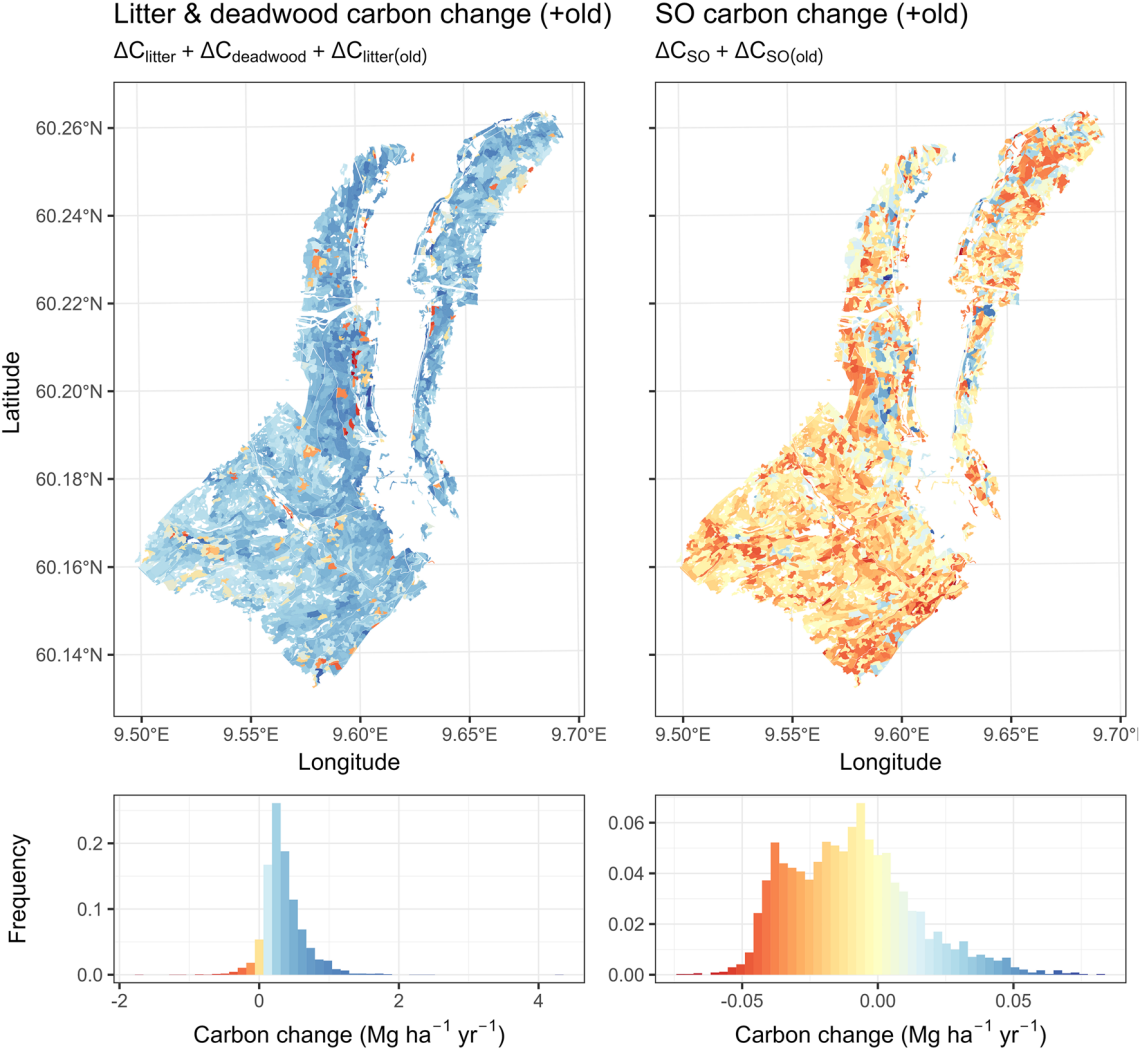
Estimated stand-level carbon change of the litter, and SO pools adding the change in the old stocks

Figure 11 shows ${\Delta C}_{litter}$ and ${\Delta C}_{SO}$ when considering the decay of approximated old stocks in 2001. Here the deadwood pool was merged into the litter pool. When old, accumulated litter was accounted for via approximated stocks in 2001, the total ${\Delta C}_{litter}$ ranged from − 1.762 to 4.253 Mg ha^−1^ yr^−1^. On average it was 0.349 Mg ha^−1^ yr^−1^. This means that while an average of 1.149 Mg ha^−1^ yr^−1^ new ${C}_{litter}$ + ${C}_{deadwood}$ has accumulated in the 15-year timeframe, 0.8 Mg ha^−1^ yr^−1^ of the old accumulation was emitted into the atmosphere or has transferred to ${C}_{SO}$. The old ${C}_{SO}$ emitted on average 0.046 Mg ha^−1^ yr^−1^; thus, the balance (${\Delta C}_{SO}$) was on average -0.01 Mg ha^−1^ yr^−1^. Unlike the litter and deadwood, the rate of ${C}_{SO}$ accumulation within the 15-year timeframe was on average lower than the emissions of old, accumulated ${C}_{SO}$. The total carbon balance, including emissions of the old soil carbon, is highly sensitive to the estimated old carbon stocks. While the rate of carbon flow within the chemical pools is assumed equal for the old and new soil carbon, the emissions in absolute terms are proportional to the stocks available each year. Here we used simulations to establish initial soil carbon stocks. Without empirical observations however it is difficult to assess how accurate this approximation is. If reliable estimates of the total carbon balance are needed, then the initial soil carbon stocks must be established using more robust estimators, preferably empirically validated [56].

Finally, the overall carbon change at the stand level ranged between − 8.435 and 4.696 Mg ha^−1^ yr^−1^, with an average of 0.708 Mg ha^−1^ yr^−1^. The change in the living biomass pools (AGB and BGB) ranged between − 7.371 and 4.071 Mg ha^−1^ yr^−1^ with an average of 0.369 Mg ha^−1^ yr^−1^, in the dead biomass pools (litter and deadwood) between − 1.762 and 4.253 Mg ha^−1^ yr^−1^ with an average of 0.349 Mg ha^−1^ yr^−1^, and in the SO between − 0.071 and 0.083 Mg ha^−1^ yr^−1^ with an average of − 0.01 Mg ha^−1^ yr^−1^. Aggregating the stand level estimated across the entire area results in a total carbon balance of 0.741 Mg ha^−1^ yr^−1^. The change in the living biomass pools was estimated at 0.405 Mg ha^−1^ yr^−1^, in the dead biomass pools 0.346 Mg ha^−1^ yr^−1^, and in the SO pool − 0.01 Mg ha^−1^ yr^−1^. Note that the aggregated estimates differ slightly from the means across stands since the forest stands are of different sizes.

**Fig. 12:**
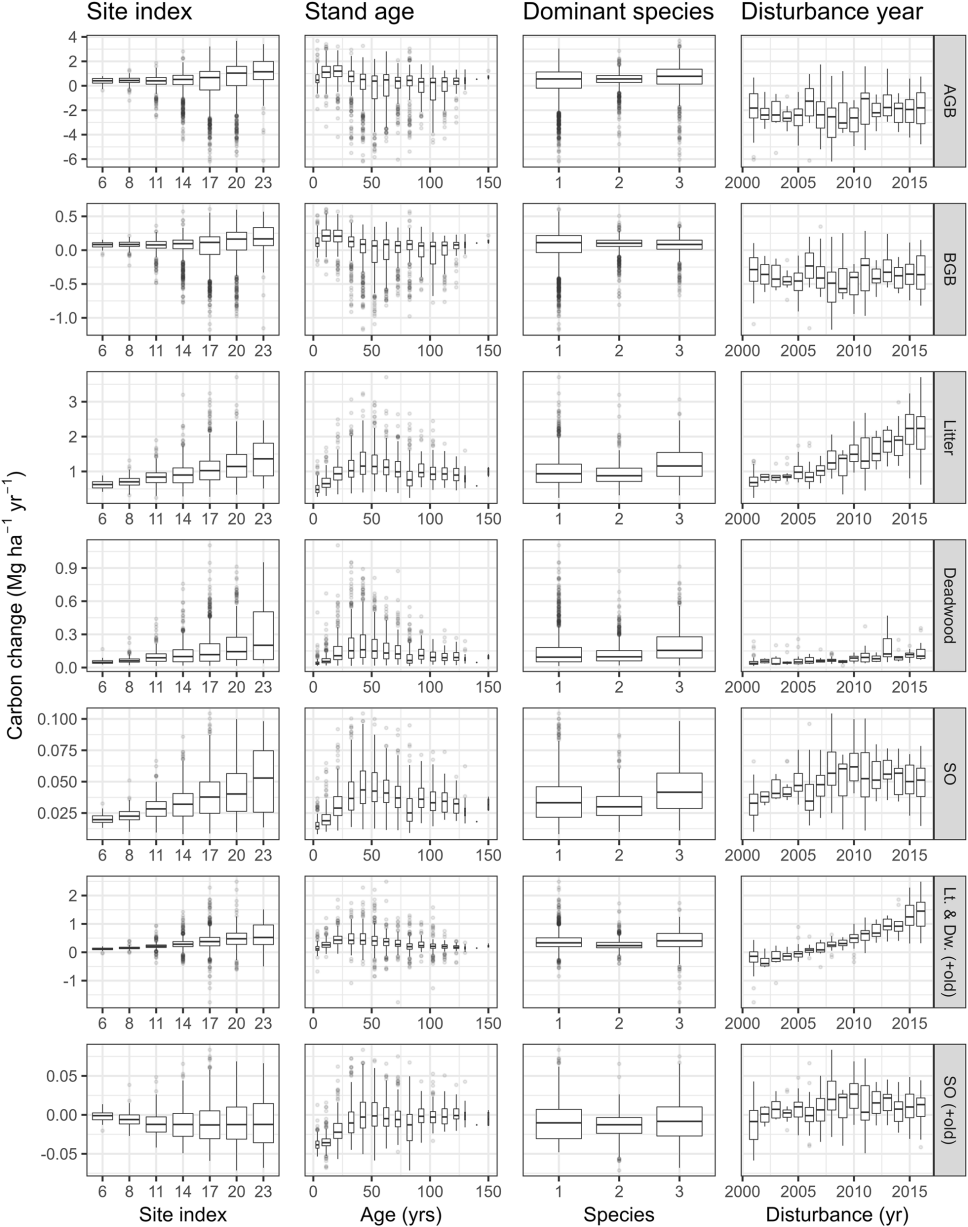
Effects of different forest stand attributes on the carbon change estimates

The effects of different forest stand properties on the estimated carbon change are shown in Fig. 12. The carbon accumulation as well as its variability in magnitude increased with forest productivity (site index). This was expected, because on the one hand, productive stands grow faster, but also loose (and transfer) higher levels of carbon when harvested. The stand age seemed to cause a trend in the soil related pools with increased accumulation up to around 50 years of age followed by a stabilization or even decline for older stands. The apparent decline is consistent with the fact that older stands are also the least productive ones (i.e., site index of 6 or 8, often pine—see Fig. 2). The dominant species did not have visible effects on the carbon change. The year of disturbance which is associated with a peak in litter input (see Fig. 7) shows clear trends for the litter and SO pools. The more recent the harvest/thinning, the more litter will be present on site at the end of the timeframe. For the SO carbon, one would expect an inverse trend, with earlier peak litter inputs having more time to decompose to SO. Disturbance however brings an additional effect of the sudden reduced or stopped stream of yearly litter. The trend that resulted from combining these effects suggested that the SO accumulation peaked 5–6 years after the disturbance.

### Errors

**Fig. 13:**
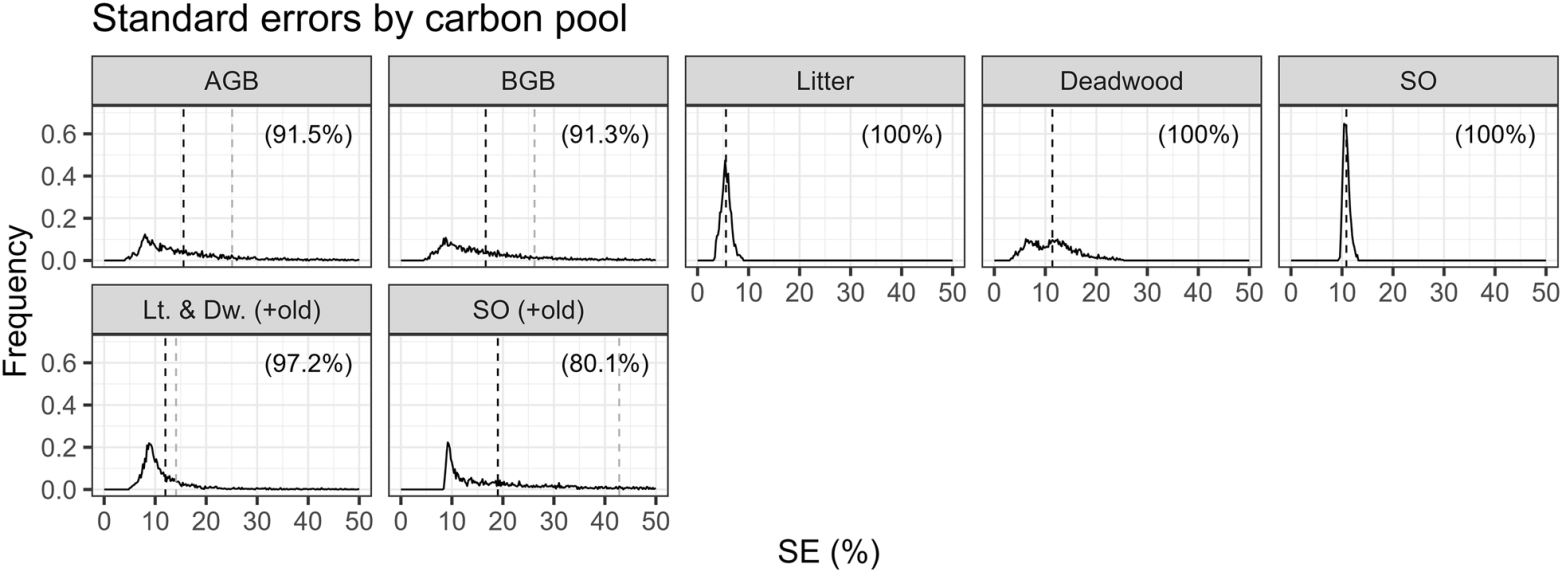
Across stands distributions of standard errors as percentage of change estimates. The x-axis is limited to values for which the change was assesed (with a 95% confidence level) to be different than 0. In parenthesis, the percentage of stands with carbon changes significantly different than 0. The black dashed line marks the mean of SEs(%) for stands with significant changes, and the gray dashed line the mean of SEs(%) for all stands. *Lt.* litter, *Dw.* deadwood

The standard errors (SEs), expressed as percentage of the estimated carbon change for each of the carbon pools are shown in Fig. 13. Extreme values, namely the 99th percentile, were filtered out. These values occur when the change estimate is near 0, and therefore the SE can be quite large if expressed as percentage of the change estimate. In Fig. 13, the x-axis extends to 50% which coincides with the limit when the 95% confidence intervals touch 0. The changes in the living biomass pools were significant for more than 90% of the stands. The carbon accumulation in the soil related pools (litter, deadwood, and SO) was significant for all stands. When the old carbon stocks were considered for the soil pools, the changes in the litter and deadwood pools were significant for more than 97% of the stands and the changes in SO for 80% of the stands. All carbon change estimates in all pools were significantly different than 0 at the level of the entire area.

The estimated SEs of ${\Delta C}_{AGB}$ and ${\Delta C}_{BGB}$ had a similar pattern and were on average 25.08% and 26.2%, respectively. For the stands with significant change the SEs were on average 15.56% and 16.63%, respectively. The skewed distributions are due to the nature of the point estimates, which are estimates of change, with a fraction of them being close to 0, and errors that are not necessary proportional to the estimates. The SE of the ${\Delta C}_{litter}$ was on average 5.56% and with a maximum of 8.78%. The SE of the ${\Delta C}_{deadwood}$ was on average 11.39% with a maximum of 25.18%. Finally, the SE of the ${\Delta C}_{SO}$ was on average 10.86% with a maximum of 13.07%. When the old soil carbon stocks were included, the SE of the litter and deadwood pools was on average 14.09% and for the SO 42.8%.

**Fig. 14:**
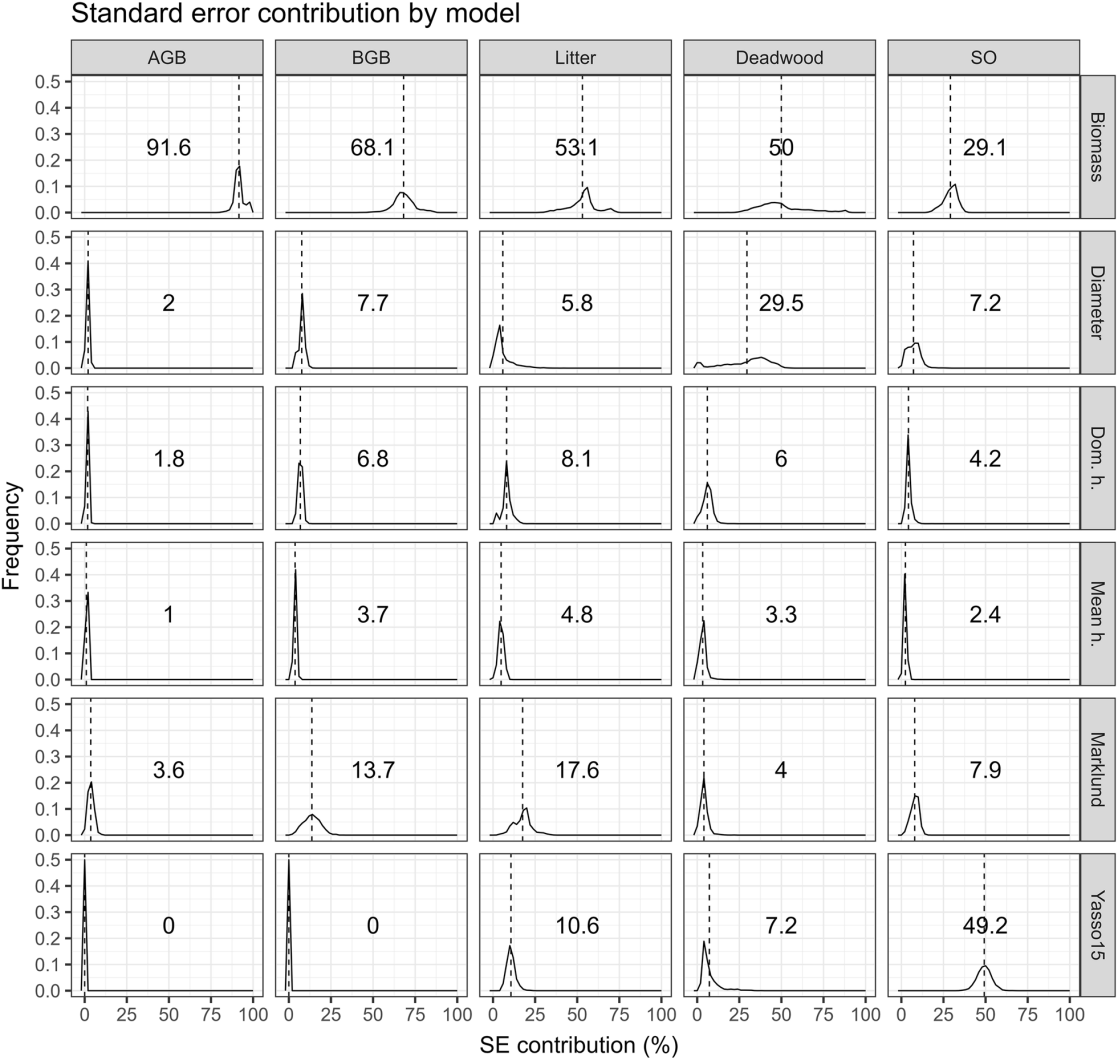
Distributions of the relative error contributed by each model to the change estimates in each pool. The dashed line marks the mean which is also printed in the center of each panel

The distribution of relative error contributions by each model are shown in Fig. 14. For AGB and BGB, the area-based biomass model was responsible for a substantial fraction of the error (91.6% for AGB and 68.1% for BGB). This was expected as this model acts as a scale factor, where AGB and BGB must sum up to the predicted total BMS, with their relative proportions being determined by the Marklund models. For BGB, 13.7% of the error was due to the Marklund models which were fitted on fewer number of observations for the root system biomass as compared to other biomass components. For the litter and deadwood pools, the area-based biomass model was still the largest error source with over 50%. Finally, for the SO pool, the Yasso15 model contributed 49.2% of the error, and area-based biomass model 29.1%. It is interesting to note that the second highest contributor was different between the litter and deadwood pools. For deadwood, the diameter model was responsible for 29.5% of the error, and for the litter pool, the Marklund model for 17.6%. This makes sense, as the litter input is differentiated among the tree components, while for the deadwood, the size of the tree is more important.

The stand level SE of the overall carbon change estimates (in all pools) was on average 18.33%. To the total SE, the living biomass pools (AGB + BGB) contributed on average 79.13%, the dead (or decomposing) biomass pools (litter + deadwood) 18.87%, and the SO pool 2%. This ranking was largely a result of the relative size of the changes in the different pools. When split by the contribution of each model the mean shares were: 80.27% biomass model, 7.34% diameter model, 3.62% dominant height model, 1.16% mean height model, 3.51% Marklund models, and 4.11% Yasso15 model. Comparing the individual model contribution is indicative of the improvement potential. The results suggest that improving the ALS based biomass model would bring the most benefit in reducing the uncertainty. This translates to increasing the field sample size which should be balanced against the cost of doing so.

Finally, the SE at the entire area level were in absolute terms: 0.14 Mg ha^−1^ yr^−1^ overall, 0.13 Mg ha^−1^ yr^−1^ for the biomass pool, 0.027 Mg ha^−1^ yr^−1^ for litter and deadwood, and 0.003 Mg ha^−1^ yr^−1^ for SO.

In this study, the error arithmetic did not include the residual errors of the models. We expect however that for the smaller stands the residual variances would have a considerable contribution. Moreover, given the homogeneity of the typical boreal forest stand we expect the residuals to be correlated for cells within the same stand, thus increasing their contribution to the SE in the form of their spatial covariances. It is however difficult to assess this type of effect without spatially intensive field observations. Nevertheless, because the study area as a whole is of substantial size (50 km^2^), it is reasonable to assume that the residual error components should have negligible effect on the overall SE estimate for the entire study and even for sub-regions within the study area, such as individual forest holdings [63]. More generally, in the lack of local validation data for the soil related pools, it is difficult to assess the chained models’ predictions, and the stand level estimates that are based on them. It is thus expected that the errors are underestimated under the effect of several different factors such as the models with unaccounted uncertainty, or the possible bias introduced by external models. Moreover, at local spatial scales such as area plots or individual forest stands, ground observations on soil carbon levels may not be sufficient in determining a spatially strict carbon balance (i.e., within a plot or stand boundary). In addition to the “vertical” fluxes that are addressed by the models of the present study, topography and rainwater create “horizontal” fluxes in soil carbon, transporting it away from the site. In this sense, our modeling approach is vertically consistent at the forest stand level, meaning that all soil carbon produced within the stand boundaries is traced through decomposition flows irrespective of any potential spatial flows. Nonetheless, a landscape-level sample of soil carbon observations would be valuable as it would enable to calibrate the stand level soil carbon estimates of change [64]. Alternatively, a similar calibration could possibly be performed using a network of atmospheric carbon flux sensors [44].

## Conclusion

We have developed an integrated methodology to estimate the changes in five important forest carbon pools taking advantage of repeated ALS surveys, a well-established methodology for forest management inventory, and existing models for allometry and soil carbon dynamics. Our results show that ALS data can be used indirectly through a chain of models to estimate soil carbon changes in addition to changes in biomass at the primary level of forest management, namely the forest stands. Having control of the errors contributed by each model, reliable inference can be made under a model-based inferential approach.

## Data Availability

The case study dataset is available on reasonable request to the authors.

## Appendix A

Yearly litter turnover

See Table 5.

**Table 5:** Turnover rates for individual biomass components

	$SW$	$RF$	$FL$	$BR$	$DB$	$RC$	$SU$	$SB$
Spruce	0	0.6	0.143	0.0125	0.0125	0.0125	0	0
Pine	0	0.6	0.33	0.027	0.027	0.027	0	0
Birch	0	0.6	1	0.025	0.025	0.025	0	0

$SW$stem wood, $RF$ fine roots, $FL$ foliage, $BR$ branches, $DB$ dead branches, $RC$ coarse roots, $SU$ stump, and $SB$ stem bark; the rates are expressed as an yearly proportion and are a result of estimated lifetime (e.g. lifetime of pine needles is approx. 3 years, thus the yearly turnover rate of 0.33). The values were compiled in Peltoniemi, Mäkipää [65] and de Wit, Palosuo [66]

## Appendix B

Chemical composition of litter

The chemical composition of individual tree components is shown for each tree species in Table 6 (spruce), Table 7 (pine), and Table 8 (birch). The chemical compartments are celluloses ($A$), sugars ($W$), wax-like compounds ($E$), and lignin-like compounds ($N$). The tree components are stem wood ($SW$), fine roots ($RF$), foliage ($FL$), branches ($BR$), dead branches ($DB$), coarse roots ($RC$), stump ($SU$), and stem bark ($SB$). The values are averages from Appendix 1 in the yasso07 manual [67].

See Tables 6, 7, 8

**Table 6:** Chemical partition of biomass components for spruce

	$A$	$W$	$E$	$N$
$SW$	0.666667	0.017949	0.002564	0.312821
$RF$	0.5508	0.1331	0.0665	0.2496
$FL$	0.4826	0.1317	0.0658	0.3199
$BR$	0.666667	0.017949	0.002564	0.312821
$DB$	0.666667	0.017949	0.002564	0.312821
$RC$	0.666667	0.017949	0.002564	0.312821
$SU$	0.666667	0.017949	0.002564	0.312821
$SB$	0.5508	0.1331	0.0665	0.2496

**Table 7:** Chemical partition of biomass components for pine

	$A$	$W$	$E$	$N$
$SW$	0.680203	0.022843	0.007614	0.28934
$RF$	0.5791	0.1286	0.0643	0.228
$FL$	0.518	0.1773	0.0887	0.216
$BR$	0.462586	0.019888	0.084075	0.433451
$DB$	0.462586	0.019888	0.084075	0.433451
$RC$	0.462586	0.019888	0.084075	0.433451
$SU$	0.680203	0.022843	0.007614	0.28934
$SB$	0.5791	0.1286	0.0643	0.228

**Table 8:** Chemical partition of biomass components for birch

	$A$	$W$	$E$	$N$
$SW$	0.715	0.015	0	0.27
$RF$	0.43395	0.19545	0.0977	0.2729
$FL$	0.43395	0.19545	0.0977	0.2729
$BR$	0.715	0.015	0	0.27
$DB$	0.715	0.015	0	0.27
$RC$	0.715	0.015	0	0.27
$SU$	0.715	0.015	0	0.27
$SB$	0.43395	0.19545	0.0977	0.2729

## Abbreviations

AGB: Above-ground biomass
ALS: Aerial laser scanning
BGB: Bellow-ground biomass
IPCC: Intergovernmental Panel on Climate Change
LULUCF: Land use, land-use change and forestry
NFI: National forest inventory
SI: Site index
SO: Soil organic (carbon)
UNFCCC: United Nations Framework Convention on Climate Change
